# Dose-Response Relationship of a Blended In-Person and Online Family-Based Childhood Obesity Management Program: Secondary Analysis of a Behavior Intervention

**DOI:** 10.2196/36770

**Published:** 2022-07-05

**Authors:** Sam Liu, Nicholas Smith, Kayla Nuss, Megan Perdew, Dimas Adiputranto, Patti-Jean Naylor

**Affiliations:** 1 School of Exercise Science, Physical and Health Education University of Victoria Victoria, BC Canada

**Keywords:** engagement, dose response, childhood obesity, web-based intervention, child, obesity, weight, web based, intervention, family, families, lifestyle, parent, parental support, healthy eating, family support, physical activity, diet, exercise, fitness, online portal

## Abstract

**Background:**

The Early Intervention Program (EIP) was a 10-week, blended, in-person and online lifestyle intervention for families with children who were off the healthy weight trajectory. The engagement pattern and the dose response of EIP have not been examined.

**Objective:**

The aims of this paper are to examine families’ engagement patterns with the EIP and to evaluate the dose-response relationship between EIP engagement patterns and physical activity and healthy eating–related outcomes at 10 weeks.

**Methods:**

Families with children (8-12 years old) who are off the healthy weight trajectory (child BMI ≥85th percentile for age and sex) were recruited. Pre- and postintervention questionnaires assessed child lifestyle behaviors, parental support behaviors, family lifestyle habits, as well as parental physical activity and healthy-eating identity. Hierarchical cluster analysis of both in-person and online components was used to classify engagement patterns. Regression analysis assessed differences in outcomes by engagement groups.

**Results:**

Two distinct clusters of engagement groups were identified (N=66), which were in-person (IP; n=40, 61%) and in-person + online (IP+; n=26, 39%) engagement. Relative to the IP group at week 10, IP+ showed a greater child moderate-to-vigorous physical activity level (1.53, SD 0.56; *P*=.008), child physical activity confidence (1.04, SD 0.37; *P*=.007), parental support for child physical activity (5.54, SD 2.57; *P*=.04) and healthy eating (2.43, SD 1.16; *P*=.04), family habits for physical activity (3.02, SD 1.50; *P*=.049) and healthy eating (3.95, SD 1.84; *P*=.04), and parental identity for physical activity (2.82, SD 1.19; *P*=.02).

**Conclusions:**

The online EIP portal complemented the in-person sessions. Additional engagement with the portal was associated with greater improvements in child physical activity and parental support behaviors, habits, and identity for physical activity.

## Introduction

The rising prevalence of childhood obesity worldwide is a major public health concern. In Canada, the combined prevalence of overweight and obesity among children and adolescents increased from 23% in 1978-1979 to 35% in 2004 [1]. Recent data suggest that the prevalence of childhood overweight and obesity has stabilized in the last decade; however, over 31% of children and adolescents (aged 6-17 years old) are still overweight (18%) or obese (13%) in Canada [1]. In 2017-2018, the prevalence of obesity among children and adolescents aged 2-19 years was 19.3% in the United States [2]. The latest data from England suggest that 25.5% of the children between the ages of 10 and 11 years were obese, and 15.4% were overweight [3]. The rising prevalence of being overweight and obesity among children in these counties has been associated with several lifestyle factors including physical inactivity, unhealthy eating patterns, and insufficient sleep [4].

Childhood obesity has been linked to several physiological and psychological consequences throughout childhood [5,6]. For example, children with obesity are more likely to be diagnosed with chronic conditions such as heart disease, which were once only identified in adults [7]. Further, obesity that develops during childhood often continues into adulthood and is associated with shorter lifespans and lower quality of life [6]. Consequently, the development of lifestyle interventions for childhood obesity treatment and prevention have become a priority for public health agencies globally.

Family-focused behavioral interventions aimed to promote a healthy lifestyle, such as regular physical activity and a healthy diet, are one of the principal approaches for the management of obesity among children [8,9]. Parental involvement is a key component in family-based interventions since parents provide their children with the necessary support for adopting a healthy lifestyle in the home environment [10,11]. Family-based interventions targeting children aged 8-12 years can be particularly effective in managing childhood obesity. Prepuberty children have potential to grow in height, which can enable them to return to healthy growth parameters in the normal course of growth if their weight is controlled [12,13]. Children in this age group can be more flexible than adults in their ability to change behaviors, because they are just beginning to develop self-regulation skills for healthy living [12]. Several randomized controlled trials have demonstrated that family-focused behavior interventions delivered in person can be effective strategies to manage childhood obesity [8,9]. However, due to the requirements of in-person interventions such as travel to a location or missing work responsibilities, their structures are often limited in flexibility and scalability [14]. Emerging evidence has indicated the inclusion of digital technologies (eg, smartphones, tablets, computers, and wearables) in conjunction with in-person, family-based interventions, which may allow researchers to increase both program outreach and provide flexibility for families [15,16].

The Early Intervention Program (EIP) implemented a blended design including both in-person and online components to promote healthy lifestyle intervention for off-trajectory children (ages 8-12 years with a BMI ≥85th percentile) and their families in British Columbia (BC), Canada. The blended intervention design can help improve program delivery flexibility [16]. The EIP curriculum integrated the Multi-Process Action Control (M-PAC) framework, and emphasized behavior change techniques such as goal setting, self-monitoring and feedback, as well as social support [17]. Intervention activities focused on behavior change skills that enabled children and their families to develop regular physical activity and healthy dietary behaviors. Our team recently evaluated the effectiveness of the EIP. Our results suggested that children in the EIP blended intervention, relative to control, significantly improved in moderate-to-vigorous physical activity (MVPA), as did parental support for healthy eating and physical activity [18].

Currently, families’ engagement patterns with the EIP program have not been examined. Both the online and in-person intervention components may influence participants’ ability to achieve the desired behavior outcomes. The relationship between intervention engagement (dose) and intervention outcome (response) is an important outcome for digital health intervention [19]. Previous studies have shown that intervention engagement was associated with improvements in physical activity and health-related outcomes [14,19]. Currently, there is a lack of studies examining the dose-response relationship for blended family-based healthy lifestyle interventions for off-trajectory children.

Thus, the study objectives were as follows: (1) to examine families’ engagement patterns with the EIP; and (2) to evaluate the dose-response relationship between EIP engagement patterns and physical activity and healthy eating–related outcomes at 10 weeks. We hypothesized that there were distinct patterns of program engagements, and participants who demonstrated a greater engagement with the intervention would show greater improvements in lifestyle behavior outcomes.

## Methods

### Study Design

This study was a secondary analysis of data from a trial evaluating the effectiveness of EIP (October 2018 to March 2019) [18] and EIP scale-up evaluation (April 2019 to September 2019). All participants enrolled in the EIP intervention during October 2018 to September 2019 were included in this study. EIP was delivered at one of the following local community centers in BC, Canada: Prince George (YMCA of Northern BC); Kelowna (YMCA of the Okanagan); Surrey (Tong Louie YMCA); Surrey (City of Surrey); Burnaby (City of Burnaby); and Greater Victoria (Westshore Recreation and Parks Society). Recruitment strategies included the following: newspaper advertisements, letters, and email blasts to provincial networks and organizations; posters and rack cards displayed in recreation centers, public community spaces, medical offices, and schools; and social media advertisements.

### Ethics Approval

Informed consent was obtained from all parents, and children were asked to complete an ascent form confirming that they understood the terms of participating in the study. Participant confidentiality was maintained throughout the study by having no participant names on any of the data. This study was approved by the University of Victoria and University of British Columbia Research Ethics Boards (BC18-024).

### Participants

The inclusion criteria were children between the ages of 8 and 12 years who are ≥85th percentile BMI for age and sex [20]. The program required the participation of at least one parent, caregiver, or legal guardian along with the children. The exclusion criteria were children with known health issues such as cardiovascular disease, mental health issues, or eating disorders; children who had a BMI of <85th percentile; and if the parent and child were unable to communicate in English.

### Early Intervention Program

EIP represents a community-based delivery model that was theoretically informed by the M-PAC framework, which emphasizes social cognitive approaches to facilitate intention formation and the adoption of action control through self-regulation, including an action control maintenance phase where a behavior becomes habitual and self-identified [17]. EIP was developed to complement the existing childhood obesity management program in BC, Canada (HealthLink BC Eating and Activity Programme for Kids: telephone-based support program for children who were overweight; Shapedown: a clinical-based program for children with BMI ≥97th percentile for age and sex).

Intervention activities were designed to instruct and support children and parents in learning about and experiencing supportive lifestyle behaviors (eg, increased physical activity), positive mental health strategies (eg, gratitude and appreciation circles), and behavior change techniques (eg, goal setting, feedback, and monitoring).

The 10-week EIP included weekly interactive in-person group sessions and online activities. A minimum of 5 families were needed to run the intervention at each program site. Group sessions were held once a week for 90 minutes and included family physical activity; child-only physical activity aiming at improving enjoyment, confidence, motivation, and fundamental movement skills; and parent-only group discussion to identify barriers and strategies for promoting healthy lifestyle behaviors. Following the in-person sessions, 10 weekly online interactive lessons were made available to the families using a web portal. The weekly online lessons complemented the in-person sessions by offering additional resources about healthy living, weekly physical activity challenges, family recipes ideas, personal diaries for family goal setting and monitoring, and an online discussion forum. Families were encouraged to complete weekly self-directed online portal activities. The in-person group session content was also made available on the portal in case families were not able to attend the weekly sessions.

### Study Procedure

Study data were collected by a research assistant at baseline and 10-week follow-up at each study site. The parents and children completed a survey prior to attending their first session and final program sessions. Demographic information (ie, ethnicity, parent education, and annual household income) was collected at baseline.

### EIP Engagement Metrics

#### In-Person Engagement

This was calculated using the total number of in-person sessions attended over the 10 possible occasions a family could attend the in-person component of the intervention. Session attendance was recorded by site facilitators if the participant was present for the entire in-person session.

#### Web Portal Log-in Frequency

The total number of log-ins consisted of the number of times the families logged onto the online EIP portal throughout the EIP program. All modules could be completed during a single log-in occasion; however, the participants were allowed to log in as many times as they wished.

#### Weekly Online Minutes

The average minutes per week a family spent logged into the portal was recorded. The average weekly time was calculated by dividing the total time by 10 (the length in weeks of the EIP program).

#### Percentage of Online Content Accessed

Data were captured for each distinct weekly lesson webpage a family accessed. A total of 111 webpages contained content regarding behavior improvements, such as strategies to improve physical activity, different healthy recipes, as well as family physical activity and healthy eating challenges.

### Child Measures

#### Physical Activity

Weekly MVPA was assessed using a child physical activity questionnaire that was based on the guidelines provided by the Physical Activity Questionnaire for Older Children (=.79) [21]. In order to determine the days per week of MVPA, children were asked to indicate how many days over the course of the previous week they were physically active for a total of at least 60 minutes, including all the time they spent doing activities that increase their heart rate or made them breathe hard.

#### Physical Activity Confidence

Physical activity confidence was measured using the Patient-centered Assessment and Counselling for Exercise questionnaire (=.75) [22]. This questionnaire included a 5-point Likert scale that assessed if a child felt confident preforming physical activity when they felt sad; whether they dedicated time to preform physical activity; whether they could maintain a commitment to physical activity when their family wanted to do another activity; whether they woke up early to perform physical activity; whether they continued to perform physical activity when they had school work; and if they still preformed physical activity despite poor weather conditions (ie, rainy or humid days).

#### Dietary Behaviors

Fruit, vegetable, and sugary sweetened beverages intake were assessed using questions from the Centre for Disease Control and Prevention Behavioral Risk Factor Surveillance System 7-day recall (intraclass correlation coefficient=0.50) [23].

### Parental Measures

#### Parental Support for Healthy Eating and Physical Activity

Three items were adapted from previous research [24,25]. The eating items were scored on a 1 (not at all) to 4 (every day), which began with the following stem: “During a typical week, how often have you or a member of your household.” The items were as follows: “Encouraged your child to eat more fruit,” “Encouraged your child to eat more vegetables,” and “Bought fruit or vegetables that you know your child likes.” The physical activity items were scores on a scale of 1 (strongly disagree) to 5 (strongly agree) and were as follows: “I watch my child play sports or participate in other activities such as martial arts or dance,” “I enroll my child in sports teams and clubs such as soccer, basketball, and dance,” and “I take my child to places where he/she can be active.”

#### Family Habits for Eating and Physical Activity

Family healthy eating and physical activity habits were measured using The Self-Report Index of Habit Strength, which included a 5-point Likert scale and questions such as the following: “preparing and eating healthy meals and snacks is something I do automatically…” and “participating in physical activity as a family is something we do without thinking” [26].

#### Parental Identity for Healthy Eating and Physical Activity

Three items, adapted from the role identity subscale of the Exercise Identity Scale measured identity for eating (α=.82) and physical activity (α=.88) [27]. The eating items were as follows: “I consider myself an individual who prepares healthy food and beverage choices;” “When I describe myself to others, I usually include my commitment to eating healthy;” and “Others see me as someone who regularly eats healthy.” The physical activity items were as follows: “I consider myself an exerciser;” “When I describe our family to others, I usually include something about our physical activities;” and “Others see us as a family that is regularly active.” Each item was scored on a scale that ranged from 1 (strongly disagree) to 5 (strongly agree).

### Data Analysis

Patterns in missing data were examined for each of the behavior outcomes separately to ensure that the data were missing completely at random. Missing data were imputed using the data using baseline observations carried forward. Hierarchical cluster analysis by means of the Ward method was used to explore EIP engagement patterns [28]. The engagement data (ie, weekly in-person attendance, frequency of online log-ins, percentage of online portal content accessed, and average weekly time spent engaging with the online EIP portal) were converted into z-scores and included in the cluster analysis. The hierarchical cluster identified the two following clusters: (1) families that mostly engaged with in-person (IP) sessions; and (2) families that engaged with both in-person and online (IP+) sessions.

Independent *t* tests were used to explore whether the baseline family characteristics differed between the patterns of engagements for continuous variables (eg, child and parent outcome measures). Chi-squared tests were used to explore differences between the engagement groups for categorical variables (eg, child ethnicity, family income, and parent education).

Linear regressions were used to compare whether child and parent outcome measures differed at follow-up between the 2 engagement groups. Each regression model was adjusted for baseline values of our dependent variable. Data were analyzed using SPSS V26.0 (IBM Corp). The statistical significance criterion was set to *P*<.05.

## Results

### EIP Engagement Patterns

The cluster analysis revealed 2 distinct engagement clusters, which were families that engaged mostly with the IP sessions (n=40) and families that engaged with the IP+ sessions (n=26). We did not observe significant baseline differences between the groups (IP vs IP+) for parent education, child ethnicity, child physical activity, child dietary behaviors, parental support for child physical activity and healthy eating, as well as physical activity and healthy eating identity and habits (*P*>.05; Table 1). However, we observed a significant difference for family income between the groups (N=55, *X*^2^_2_=6.2; *P*=.02). Specifically, families with higher income (more than CAD $59,000 [US $45,000]) were more likely to engage with both in-person and the online portal compared with families with lower income (less than CAD $59,000 [US $45,000]).

Over the 10-week period, the mean in-person session attendance percentage for both groups was 8.1, 81.03% (SD 1.54). The mean EIP online portal engagement for both groups for log-in frequency was 3.29 (SD 2.98) times, mean weekly portal engagement minutes was 14.57 (SD 13.47) minutes, and families accessed on average 22.19% (SD 21.74) of the online portal content. The number of in-person sessions attended did not vary significantly between these 2 groups (*P*>.05). However, engagement of the online portal did vary significantly between the groups (*P*<.05). The IP+ group showed a greater number of online portal log-ins, weekly engagement minutes, and percentage of content accessed (Table 2).

**Table 1:** Baseline characteristics by EIP^a^ engagement patterns (N=66).

Characteristics			Engaged with in-person sessions (n=40)		Engaged with both in-person and online sessions (n=26)		*P* value
**Ethnicity, n (%)**							.63
	White	17 (42)		14 (54)			
	Indigenous	3 (8)		3 (12)			
	Asian^b^	8 (20)		4 (15)			
	Other^c^	2 (5)		2 (7)			
	Multi-ethnicities^d^	10 (25)		3 (12)			
**Parent education, n (%)**							.30
	High school diploma	5 (14)		6 (25)			
	2-year college	16 (43)		12 (52)			
	University	7 (19)		3 (14)			
	Graduate degree	9 (24)		2 (9)			
**Household income^e^ (US $), n (%)**							.01
	<45,000	18 (53)		4 (19)^f^			
	>45,000	16 (47)		17 (81)^f^			
60 min of MVPA^g^ (days per week)			3.28 (2.28)		3.58 (2.41)		.61
Child physical activity confidence^h^			2.62 (1.34)		3.08 (1.27)		.17
Child fruit intake, times per day in a typical week (SD)			2.10 (1.17)		2.58 (1.31)		.34
Child vegetable intake, times per day in a typical week (SD)			1.82 (1.42)		2.01 (1.30)		.88
Child sugary drink intake, times per day in a typical week (SD)			1.47 (1.03)		1.75 (1.25)		.34
Parental physical activity support^i^			21.07 (5.90)		21.61 (3.38)		.76
Parental support for healthy eating^j^			10.49 (2.03)		9.93 (1.13)		.20
Family healthy eating habit^k^			11.85 (5.17)		12.58 (3.18)		.52
Family physical activity habit^l^			9.70 (4.90)		10.88 (4.24)		.32
Parental healthy eating identity^m^			10.17 (3.30)		10.07 (2.30)		.89
Parental physical activity identity^n^			7.78 (3.3)		8.67 (2.68)		.30

^a^EIP: Early Intervention Program.

^b^Asian: South Asian, East Asian, Chinese, and Southeast Asian.

^c^Other: Black and Latin American.

^d^prefer not to answer, n=6.

^e^prefer not to answer, n=11.

^f^Post-hoc chi-square significant group difference (*P*<.05).

^g^MVPA: moderate-to-vigorous physical activity.

^h^Higher value represents higher physical activity confidence (scale: 1-5).

^i^Higher value represents higher parental physical activity support (scale: 5-25).

^j^Higher value represents higher parental support for healthy eating (scale: 4-12).

^k^Higher value represents higher family health eating habit (scale: 5-15).

^l^Higher value represents higher family physical activity habit (scale: 5-15).

^m^Higher value represents higher parental healthy eating identity (scale: 0-12).

^n^Higher value represents higher parental physical activity identity (scale: 0-12).

**Table 2:** EIP^a^ engagement profile (N=66).

EIP engagement metrics		Engaged with in-person sessions (n=40)	Engaged with both in-person and online sessions (n=26)	*P* value
Percent of in-person attendance, n (%, SD)		7.9 (79.8, 1.60)	8.5 (84.54, 1.32)	.13
**Online engagement**				
	Total number of log-ins, mean (SD)	1.37 (1.12)	6.23 (2.45)	<.001
	Average weekly time spent online, min (SD)	6.23 (2.49)	26.85 (13.16)	<.001
	Core webpages accessed, % (SD)	7.26 (6.24)	45.16 (16.22)	<.001

^a^EIP: Early Intervention Program.

### The Relationship Between EIP Engagement and Intervention Outcomes

Child physical activity (MVPA), and physical activity confidence at follow-up were significantly higher in the IP+ group than in the IP group (*P*<.05). We did not observe a significant between-group difference in dietary behaviors (Table 3). Parental support for child physical activity and healthy eating, as well as family habits for healthy eating and physical activity were significantly higher in the IP+ than the IP group (*P*<.05). Family physical activity identity was also significantly higher in the IP+ group than in the IP group (*P*<.05). No significant difference between the groups was observed for family healthy eating identity (Table 3).

**Table 3:** Comparison of family behavior outcomes in the EIP^a^ engagement patterns follow-up.

EIP outcomes	In-person + online vs in-person sessions, B^b^ (SE)	*P* value
Child physical activity (days per week reaching 60 min of MVPA^c^)	1.53 (0.56)	.008
Child physical activity confidence	1.04 (0.37)	.007
Child fruit intake (times per day in a typical week)	0.73 (0.45)	.11
Child vegetable intake (times per day in a typical week)	0.05 (0.36)	.88
Child sugary drink intake (times per day in a typical week)	–0.26 (0.25)	.16
Parental physical activity support	5.54 (2.57)	.04
Parental support for healthy eating	2.43 (1.16)	.04
Family healthy eating habit	3.95 (1.84)	.04
Family physical activity habit	3.02 (1.50)	.049
Parental healthy eating identity	2.19 (1.30)	.1
Parental physical activity identity	2.82 (1.19)	.02

^a^EIP: Early Intervention Program.

^b^B: linear regression models adjusted for baseline variable.

^c^MVPA: moderate-to-vigorous physical activity.

## Discussion

### Principal Findings

Family-based lifestyle interventions can be an effective way to promote regular physical activity and healthy eating among families with children who are off the healthy weight trajectory. The blended in-person and online delivery model can help further improve program flexibility and scalability. This is one of the first studies to explore engagement patterns and the dose-response relationship of a blended family-based lifestyle intervention designed for families who are off the healthy weight trajectory. Our findings assist in understanding the impact of program engagement on intervention outcome and ways to improve intervention engagement.

Our results suggested that engagement with the in-person component of the EIP was high among both groups (IP and IP+). Engagement with the online component of the intervention was the distinguishing factor between the 2 groups. The additional online engagement (IP+) resulted in greater improvements than the IP group in child physical activity behaviors, parental support for child physical activity and dietary behaviors, as well as family physical activity and dietary habits and identity. This observed dose-response relationship between intervention usage and outcomes was reported in previous online studies among children and adolescents [19,29]. Our results contribute to this field by demonstrating the potential complementary effects of online intervention with in-person intervention for family-based lifestyle programs.

Families that engaged with both the online portal and the in-person EIP (IP+) added almost 1.5 days per week of at least 60 minutes of child MVPA compared with families who mostly engaged with the in-person group (IP). A number of studies have reported that child physical activity level is associated with parental support behaviors, family physical activity habits, and parental exercise identity [10,30]. Thus, it is not surprising that compared to the IP group, the IP+ group showed a greater improvement in parental support for child physical activity, family habits, and parental physical activity habits. The EIP was designed based on the M-PAC framework to strengthen behavior intention formation (eg, the physical and mental health benefits of physical activity as well as parental support behaviors) and promote behavior maintenance (restructuring the physical and social environment to create opportunities for physical activity, habit formation, and identity formation). The online component of the EIP offered families additional opportunities to engage in physical activity together through various challenges and activities in their local community (eg, outdoor games and geotagging). Some studies indicate that parents’ opportunity to coparticipate in physical activity with their children is associated with an increase in parental support [31]. We speculate, then, that the suggestions provided for family physical activity in the EIP online portal influenced parent support for physical activity.

Furthermore, according to the M-PAC framework, the improvements in physical activity identity are related to physical activity participation. Specifically, repeated participation in physical activity may improve the perception of the ability to engage in the behavior and enhance the participant’s perception of their commitment to the behavior [32]. Both of these constructs support continued participation in physical activity, which, in turn, promotes increased physical activity identity. Similarly, in the early stages of physical activity engagement, repeated participation builds habit formation, which then increases the probability of repeated engagement [29]. Since the EIP program’s online component provided resources and opportunities for at-home family physical activity, the families were able to review those resources and actively engage in the behavior. As such, we associate the increases in parental identity and habit with family physical activity engagement at home.

We found that increased online portal engagement was not associated with improvements in child dietary outcomes, but we did detect an increase in parental support for healthy eating and habit and parental identity for healthy eating. As with the physical activity psychological constructs, we associate these increases with the additional portal resources engagement such as family nutrition challenges and recipes. We also anticipate that the lack of significant change in actual child-eating behavior may be due to ceiling and floor effects, whereby children were consuming an adequate level of fruit and vegetables and few sugary drinks (ie, none to 1-3 sugary drinks in the past 7 days) at baseline, thus reporting minimal change at follow-up [33].

Additionally, we found that baseline family income was significantly associated with online portal engagement. Sociodemographic characteristics such as socioeconomic status were associated with lower computer literacy skills and access, resulting in lower engagement with digital health interventions [34]. According to the Digital Health Engagement Model, there are several potential ways to improve engagement with the digital interventions [35]. For example, providing tutorials on how to use the online portal during the first in-person session may help families familiarize themselves with the available online tools and the additional resources. Furthermore, ensuring the web portal is accessible to mobile phones can help provide additional ways to access the program when a computer is not available. These changes to the EIP may further improve the scalability and flexibility of EIP delivery.

### Limitations

There are several limitations to this study. First, our findings may have limited generalizability due to the small sample size. Additionally, the portal usage metrics reported may not accurately reflect the participants’ actual usage within the portal. For example, weekly portal minutes are reflective of the number of minutes the participants view the portal, but it does not show whether the participants were viewing the portal, or it may also be possible that the page was left open on the desktop. The long-term effect of the program remains unclear. Lastly, the quality of interaction the participants had with the in-person sessions was not monitored.

### Conclusion

We identified 2 main types of engagement patterns (IP and IP+) with the blended family-based healthy lifestyle intervention for children who are off the healthy weight trajectory. Engagement level with the in-person component of the program remained high in both groups. However, relative to the in-person engagement group (IP), families that engaged with both in-person and online (IP+) improved child physical activity level, child MVPA, child physical activity confidence, parental support for child physical activity and healthy eating, family habits for physical activity and healthy eating, as well as parental identity for physical activity. There were no significant changes between the groups for child dietary outcomes, which may be attributed to a ceiling effect in fruits and vegetable consumption and a floor effect in sugary drink consumption. This study suggests the benefit of adding an online component to an in-person family-based childhood obesity intervention.

## Abbreviations

BC: British Columbia
EIP: Early Intervention Program
IP+: in-person + online
IP: in-person
M-PAC: Multi-Process Action Control
MVPA: moderate-to-vigorous physical activity
